# Efficacy of Remote Health Monitoring in Reducing Hospital Readmissions Among High-Risk Postdischarge Patients: Prospective Cohort Study

**DOI:** 10.2196/53455

**Published:** 2024-09-13

**Authors:** Hui-Wen Po, Ying-Chien Chu, Hui-Chen Tsai, Chen-Liang Lin, Chung-Yu Chen, Matthew Huei-Ming Ma

**Affiliations:** 1 Department of Nursing National Taiwan University Hospital Yunlin Branch Douliou Taiwan; 2 Biomedical Technology and Device Research Labs Industrial Technology Research Institute Hsinchu Taiwan; 3 Department of Internal Medicine National Taiwan University Hospital Yunlin Branch Douliou, Yunlin Taiwan; 4 Department of Emergency Medicine National Taiwan University Hospital Yunlin Branch Douliou Taiwan

**Keywords:** telemonitoring, discharge planning, case manager, hospital readmission, telehealth, remote healthcare, high-risk, post-discharge, respiratory disease, respiratory diseases, cardiovascular disease, cardiovascular diseases, case management, patient education, readmission, health status tracking, care guidance, medical advice, male, men, older adult, older adults, elder, elderly, older person, older people, home monitoring, physiological signal, physiological signals, mobile phone

## Abstract

**Background:**

Patients with respiratory or cardiovascular diseases often experience higher rates of hospital readmission due to compromised heart-lung function and significant clinical symptoms. Effective measures such as discharge planning, case management, home telemonitoring follow-up, and patient education can significantly mitigate hospital readmissions.

**Objective:**

This study aimed to determine the efficacy of home telemonitoring follow-up in reducing hospital readmissions, emergency department (ED) visits, and total hospital days for high-risk postdischarge patients.

**Methods:**

This prospective cohort study was conducted between July and October 2021. High-risk patients were screened for eligibility and enrolled in the study. The intervention involved implementing home digital monitoring to track patient health metrics after discharge, with the aim of reducing hospital readmissions and ED visits. High-risk patients or their primary caregivers received education on using communication measurement tools and recording and uploading data. Before discharge, patients were familiarized with these tools, which they continued to use for 4 weeks after discharge. A project manager monitored the daily uploaded health data, while a weekly video appointment with the program coordinator monitored the heart and breathing sounds of the patients, tracked health status changes, and gathered relevant data. Care guidance and medical advice were provided based on symptoms and physiological signals. The primary outcomes of this study were the number of hospital readmissions and ED visits within 3 and 6 months after intervention. The secondary outcomes included the total number of hospital days and patient adherence to the home monitoring protocol.

**Results:**

Among 41 eligible patients, 93% (n=38) were male, and 46% (n=19) were aged 41-60 years, while 46% (n=19) were aged 60 years or older. The study revealed that home digital monitoring significantly reduced hospitalizations, ED visits, and total hospital stay days at 3 and 6 months after intervention. At 3 months after intervention, average hospitalizations decreased from 0.45 (SD 0.09) to 0.19 (SD 0.09; *P*=.03), and average ED visits decreased from 0.48 (SD 0.09) to 0.06 (SD 0.04; *P*<.001). Average hospital days decreased from 6.61 (SD 2.25) to 1.94 (SD 1.15; *P*=.08). At 6 months after intervention, average hospitalizations decreased from 0.55 (SD 0.11) to 0.23 (SD 0.09; *P*=.01), and average ED visits decreased from 0.55 (SD 0.11) to 0.23 (SD 0.09; *P*=.02). Average hospital days decreased from 7.48 (SD 2.32) to 6.03 (SD 3.12; *P*=.73).

**Conclusions:**

By integrating home telemonitoring with regular follow-up, our research demonstrates a viable approach to reducing hospital readmissions and ED visits, ultimately improving patient outcomes and reducing health care costs. The practical application of telemonitoring in a real-world setting showcases its potential as a scalable solution for chronic disease management.

## Introduction

Health is a fundamental human right, and health care services are crucial for maintaining well-being. By 2030, the World Health Organization (WHO) aims to achieve universal health coverage, ensuring equitable access to comprehensive and quality care throughout the life of a person, regardless of financial means or socioeconomic status [1,2]. However, reaching this goal requires significant reform of existing health care systems and delivery models. Quality health care involves providing timely and appropriate treatments tailored to individual needs, while minimizing risks and conserving resources [3]. Despite these aspirations, individuals in economically disadvantaged regions face significant barriers to accessing high-quality medical care. These challenges include a severe shortage of health care providers, with only 14.3% to 44.3% of the required workforce available; inadequate diagnostic accuracy, ranging from 34.0% to 72.2%; and suboptimal adherence to clinical protocols, with compliance rates between 22.0% and 43.8% [1].

The lack of health care resources exacerbates the difficulty of maintaining continuous care for patients before and after acute illnesses or injuries. The WHO has identified two major challenges for these regions: (1) achieving “patient-centered health care” and (2) ensuring “health care continuity” [4]. These global challenges are also evident in the rural areas of Taiwan, a country known for its comprehensive health insurance system. In these regions, modern and seemingly costly information communication technology and telemedicine hold significant potential to improve health care quality. The growing older adult population, with its complex health needs, has increased the demand for critical care. Patients previously treated in intensive care units (ICUs) for conditions such as heart failure or respiratory distress are at high risk of relapse or deterioration after discharge. Preventing postdischarge regression and promptly identifying complications are crucial for both medical practice and public health [5].

The transition of ICU patients to general wards often requires prolonged hospital stays due to concerns about postdischarge exacerbations. As patients near the end of their hospital stay, the focus often shifts to ensuring physiological stability. This issue is particularly relevant globally in the context of aging populations, population mobility, and situations where primary care decision-makers are not close to older adult patients [6].

Currently, the benchmarks, metrics, methodologies, and benefits of home monitoring or telemonitoring using portable devices, wearable devices, and the internet of things remain unclear. While home monitoring for stable chronic patients may not show significant advantages due to the low likelihood of adverse events, the predictive value of various physiological data, bodily signals, and patient-reported symptoms in the early postdischarge period is not well understood [7-10].

Building on the intelligent home care system, our study focuses on managing high-risk patients during the postdischarge phase. This includes those who received ICU treatment, high-risk discharges, and patients with thoracic and cardiovascular conditions with high readmission rates. The primary goal is to monitor disease control status, medication adherence, vital sign changes, and self-care ability. We are also conducting a comprehensive prospective analysis of factors contributing to recurrent readmissions among high-risk patients and administering follow-up questionnaires to identify risk factors for predicting readmission probabilities. Our overarching aim is two-fold: (1) to reduce postdischarge readmission rates among high-risk patients and (2) to address the digital divide challenges encountered in implementing intelligent home care devices.

## Methods

### High-Risk Discharged Patient Home Care Network

**Figure 1:**
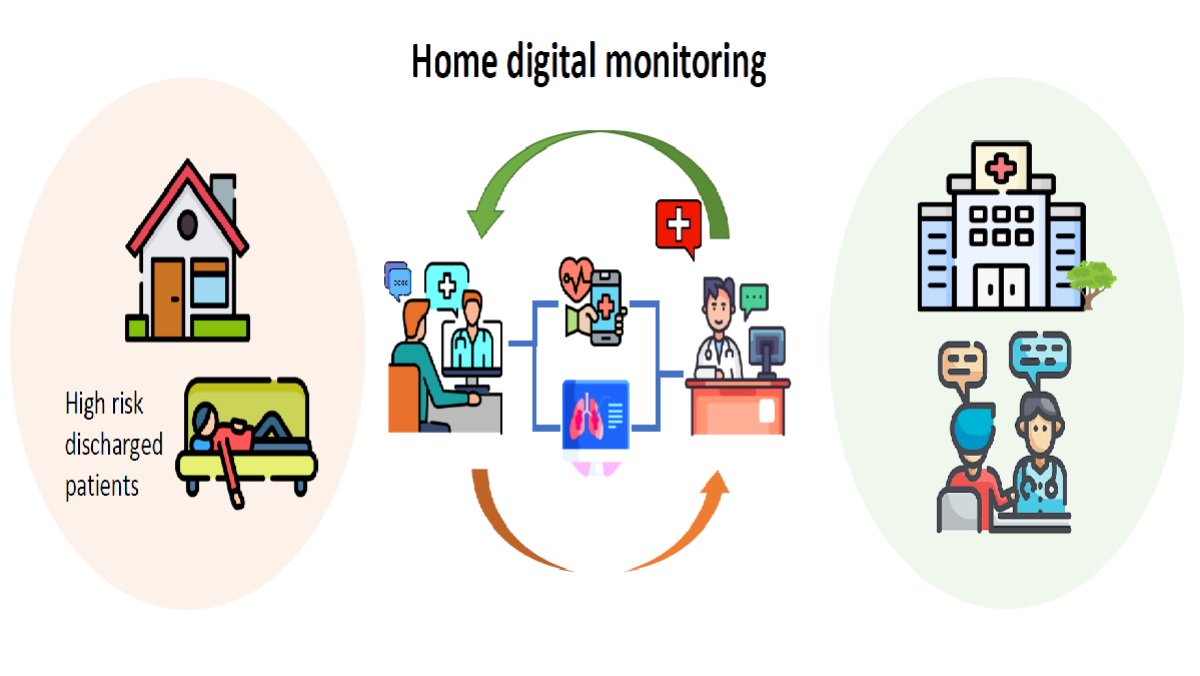
Efficient home monitoring for high-risk discharged patients.

Based at National Taiwan University Hospital Yunlin Branch, the telehealth smart discharge care network (iCARE) was established. Network members include attending physicians and case managers from the National Taiwan University Hospital Yunlin Branch, relevant patients and their family members, as well as primary care physicians in Yunlin. Case managers are responsible for coordinating the home monitoring process for high-risk discharged patients, with a monitoring period of 2-8 weeks. Primary care physicians visit the patients before discharge and actively participate throughout the monitoring process. After discharge, patients input and upload disease-related symptoms and cardiopulmonary physiological parameters through home monitoring devices. Case managers schedule regular video consultations with patients and their caregivers to inquire and record information. When necessary, they can contact the collaborative care team, including the National Taiwan University Hospital Yunlin Branch and primary care physicians. In response to changes in the patient’s condition, members of the collaborative care team, including primary care physicians or hospital case managers and physicians, can provide verbal guidance to the patients or their caregivers. If necessary, the primary care physician can visit the patient for further assessment and management. The objective is to ensure that patients can smoothly transition back to the community and prevent readmissions (Figure 1).

Case managers oversee 2-8 weeks of home monitoring for high-risk discharged patients, collaborating with primary care physicians. Patients use monitoring devices to input data. Case managers schedule video consultations and contact the care team, including primary care physicians and the hospital. The team offers guidance and in-person visits when needed to ensure a smooth community transition and reduce readmissions.

The primary outcomes of this study were the number of hospital readmissions and ED visits within 3 and 6 months after intervention. The secondary outcomes included the total number of hospital days and patient adherence to the home monitoring protocol.

### The Artificial Intelligence of Things–Based Home Surveillance and Intelligent Case Management

Hospital case management and discharge planning have been implemented for many years, and the effectiveness of case management and discharge planning can generally be reflected in terms of hospitalization days and readmission rates, indicating the quality of health care. Previous research has shown that disease-related factors contribute to prolonged hospital stays. Studies have also found higher readmission rates among patients with acute coronary syndrome, heart failure, pneumonia, and chronic obstructive pulmonary disease (COPD). However, besides the disease itself, there is limited clinical information available for analysis to predict or plan discharge preparation services. In this study, retrospective research was conducted using the electronic medical record database of National Taiwan University Hospital Yunlin Branch, including basic information, such as gender, age, diagnosis department, medication status, and primary caregivers. High-risk discharged patients were defined by criteria including activities of daily living score below 60, presence of tubes, pressure injuries or nonclean wounds, poor chronic disease control, readmission within 14 days due to the same or related factors, economic issues, inadequate primary caregiver capability, and living alone. The goal was to understand the probability of prolonged hospital stays and readmissions after discharge and to analyze relevant risk factors.

Through home monitoring and communication devices, an “intelligent case management” system was established. Symptoms, cardiopulmonary signals, and other relevant physiological parameters of high-risk patients at discharge were measured regularly using various physiological monitoring devices connected to home mobile devices. A home monitoring model was established to track disease-related changes and physiological trends, as well as the progress of care, through the management platform. Case managers supervised and coordinated the monitoring process, keeping important members of the collaborative care team and family members informed. If necessary, the collaborative care team provided care guidance and medical advice based on changes in the condition of the patient.

### Definition and Selection of High-Risk Patients

The target population includes patients diagnosed with acute coronary syndrome, heart failure, pneumonia, and COPD, or those who have received treatment in the ICU of the National Taiwan University Hospital Yunlin Branch and have been transferred to general acute wards. Before discharge, these patients are assessed by ward nurses or discharge planning case managers. Patients are classified as high-risk if they meet the following criteria: (1) exceeding the average length of stay for the specific disease or condition—patients who have been hospitalized for a duration longer than the average length of stay typically required for their diagnosed condition; (2) exceeding a specific number of days of hospitalization based on experience—patients whose hospitalization period surpasses a certain threshold determined by clinical experience and hospital protocols; and (3) meeting the service requirements based on high-risk screening—patients who are identified as high-risk through a screening process that evaluates their need for postdischarge services, considering factors such as the severity of illness, comorbidities, and potential for readmission. Common indicators for high-risk screening include (1) functional assessment score below 60 on the activities of daily living scale, (2) presence of tubes or catheters, (3) wounds or ulcers, (4) poor control of chronic conditions, (5) readmission within 14 days due to the same condition, (6) economic issues, (7) insufficient capabilities of the primary caregiver, and (8) living alone.

The following situations indicate high-risk patients at discharge: (1) patients with cardiovascular or pulmonary diseases complicated by acute respiratory failure or heart failure requiring admission to the ICU; (2) patients who need to use antihypertensive medications, oxygen equipment, or positive pressure ventilators at home after discharge; (3) patients with a lung function of forced expiratory volume 1% <50% or significant symptoms (modified Medical Research Council [mMRC]≥2 or COPD Assessment Test [CAT]≥10); (4) patients with a left ventricular ejection fraction <40% or significant symptoms (mMRC≥2); (5) patients with a BMI<16 requiring active nutritional care intervention.

For high-risk patients with cardiovascular and pulmonary diseases receiving home care, the following indicators should be monitored, and the corresponding monitoring items and parameters should be uploaded to the internet or cloud: (1) patient-reported symptoms, including chest tightness, cough, sputum production, breathing condition, and sleep quality; (2) vital signs, including body temperature, heart rate, respiratory rate, blood pressure, blood oxygen level, and level of consciousness; (3) respiratory and heart sound monitoring, including assessing for the presence of rales or increased wheezing; (4) electrocardiogram, peak expiratory flow rate, speech volume, and speech rate; (5) basic physical examination, including assessing general appearance, range of motion in limbs, and peripheral circulation; (6) basic care skills, including feeding, repositioning, percussion on the back, chest drainage posture, hygiene for bowel and bladder management, and bathing or body cleaning; and (7) verification of other care records, including confirming the depth and size of pressure ulcers or wounds and the healing process, exercise or rehabilitation activities, dietary content, and intake and output measurements.

In our study, the historical controls consisted of patients who met the same eligibility criteria as the intervention group but were discharged from the hospital before the implementation of the home digital monitoring intervention. These patients were selected from hospital records spanning a comparable time period before the intervention phase of the study. By using these historical controls, we were able to compare the health care outcomes of patients who received standard postdischarge care with those who received the enhanced monitoring and support provided by our intervention.

### Surveying the Correlation of the Digital Divide Through a Questionnaire

The study was conducted at the National Taiwan University Hospital Yunlin Branch. Recruitment took place between July and October 2021. Eligible high-risk patients were identified and enrolled before their discharge. The study targeted patients diagnosed with acute coronary syndrome, heart failure, pneumonia, and COPD, or those who had received treatment in the ICU and were transferred to general acute wards. The recruitment process involved screening patients based on specific high-risk criteria, including prolonged hospital stays and poor control of chronic conditions. Primary caregivers were involved in the education and monitoring process, ensuring comprehensive postdischarge care.

Using a closed-ended questionnaire, factors contributing to the digital divide, such as age, literacy level, education level, and proficiency in using smart devices, will be investigated to gain a better understanding of the occurrence rate of barriers in device usage, image recognition, questionnaire completion, and data transmission during the learning and utilization process (Multimedia Appendix 1). This information will serve as a basis for improvement, aiming to reduce obstacles encountered by individuals and enhance their willingness to use digital tools.

### Ethical Considerations

The study was conducted following the ethical principles outlined in the Declaration of Helsinki. The Institutional Review Board (IRB) of the National Taiwan University Hospital approved this study (IRB 202103102RINC). All procedures performed were in accordance with the ethical standards of the institutional and national research committee.

Informed consent was obtained from all individual participants included in the study. For secondary analyses of the data, the original informed consent allowed for such analyses without requiring additional consent. This was confirmed by the IRB.

The data collected in this study were deidentified to protect participant privacy. The information of the participants was anonymized, ensuring confidentiality. All data were stored securely and accessed only by authorized personnel for research purposes.

Participants were compensated for their time and effort. Each participant received a compensation of NT $500 (approximately US $18) per follow-up session. This compensation was provided to cover any incidental expenses incurred due to participation in the study.

No images included in the paper or Multimedia Appendix 1 identify individual participants. All images were carefully reviewed to ensure that no identifiable information was visible. In cases where images were necessary, consent was obtained from the individuals involved.

### Statistical Analysis

Statistical analyses were performed using paired 2-tailed *t* tests to compare the preintervention and postintervention metrics. Average hospitalization rates, ED visits, and total hospital days were calculated for each patient before and after the intervention period. All analyses were performed using SPSS computer statistical software (version 25, IBM Corp). A *P* value less than .05 was considered statistically significant.

## Results

### Impact of Home Digital Monitoring on Reducing Hospitalizations and ED Visits in High-Risk Postdischarge Patients

Between July and October 2021, a cohort of 41 patients underwent initial eligibility screening. Among these, 93% (n=38) were male. In terms of age distribution, 46% (n=19) were aged 41-60 years, while an equal percentage of 46% (n=19) were aged 60 years or older. The findings of this study revealed that the implementation of home digital monitoring as an intervention led to a significant reduction in various health care metrics within 3 and 6 months after intervention.

A comparative analysis between the periods before and after 3 months of intervention demonstrated a decrease in mean hospitalizations from 0.45 (SD 0.09) to 0.19 (SD 0.09; *P*=.03), a remarkable decline in mean ED visits from 0.48 (SD 0.09) to 0.06 (SD 0.04; *P*<.001), and a decrease in mean hospital days from 6.61 (SD 2.25) to 1.94 (SD 1.15; *P*=.08; Table 1).

**Table 1:** The impact of intervention on health care utilization: a 3- and 6-month analysis.

Impact	3 months					6 months			
	Before, mean (SD)	After, mean (SD)	Change (%)	*P* value	Before, mean (SD)		After, mean (SD)	Change (%)	*P* value
Hospitalization (n)	0.45 (0.09)	0.19 (0.09)	–57.8	.03	0.55 (0.11)		0.23 (0.09)	–58.2	.01
ED visit (n)	0.48 (0.09)	0.06 (0.04)	–87.5	<.001	0.55 (0.11)		0.23 (0.09)	–58.2	.02
Hospital stay (days)	6.61 (2.25)	1.94 (1.15)	–70.7	.08	7.48 (2.32)		6.03 (3.12)	–19.4	.73

Furthermore, the impact of the intervention remained evident after 6 months. The average number of hospitalizations decreased from 0.55 (SD 0.11) to 0.23 (SD 0.09; *P*=.01), and the average ED visits reduced from 0.55 (SD 0.11) to 0.23 (SD 0.09; *P*=.02). However, the mean hospital stay days showed a less pronounced change, shifting from 7.48 (SD 2.32) to 6.03 (SD 3.12; *P*=.73; Table 1).

### Educational Background of the Participants and Technology Usage

The majority of our participants had educational backgrounds of elementary school or junior high school, accounting for 71% (29/41). In their daily lives, 93% (38/41) of the participants used smartphones, while only 7% (3/41) frequently used other electronic devices in addition to their smartphones. Regarding self-care, 49% (20/41) of the participants indicated that they would watch television programs related to their illnesses but would not actively seek out health-related information. Only 7% (3/41) of individuals actively and regularly read articles related to health, while the rest would only watch television programs occasionally.

A total of 85% (35/41) of participants were able to operate the devices independently. Among those who performed the measurements, 95% (39/41) reported they could do so smoothly. However, 5% (2/41) mentioned that operating the electrocardiogram took more time. The difficulties they encountered included forgetting the steps, failure of the device to display a signal after attachment, and the need for additional skin preparation.

Based on these insights, we concluded that the middle-aged to older adult participants, despite their limited educational backgrounds, were generally willing to try and accept new emerging care models such as telecare. Their familiarity with smartphones and exposure to health-related information through television facilitated this transition. However, the effectiveness of the intervention was influenced by their initial hesitance and the challenges they faced in actively seeking health information.

### Impact of Participant Attitudes on the Intervention

To better understand how participant attitudes toward health information and technology usage impacted the effectiveness of the intervention, we conducted interviews with a subset of participants. These interviews revealed several key themes: (1) usage of the built-in assistance information or software of the software; (2) clarity of assistance information or software interface; (3) was the assistance information helpful to you? and (4) are you satisfied with the experience of using this care package?

Reasons for unsuccessful enrollment and data collection obstacles are outlined (Table 2). Interviews were conducted to understand why some individuals declined remote medical care. Most hesitant participants lack interest in health information, have concerns about device usage, worry about equipment damage, and find postdischarge home monitoring inconvenient due to work commitments. They resist scheduled video consultations with doctors, believing it is time-consuming, and rely on regular in-person visits and medication. Attitudes vary from negative to positive, with common traits such as older age, lack of assistance at home, and resistance to lifestyle changes.

**Table 2:** Reasons for refusing remote medical care.

Reasons	Participants (N=41), n (%)
Aged 75 years or older with children not coresiding, unfamiliar with 3C technology	12 (29)
Older adults, aged 75 years or older, unfamiliar with 3C technology, no interest to learn	10 (25)
Physically well, relying on medication, no discomfort	10 (25)
Employed, confident in self-monitoring, no scheduled video consultation	6 (14)
Physically limited, no device use, no family help	1 (3)
Willing children, individual resists continuous monitoring	2 (4)

## Discussion

This study aimed to determine the efficacy of home telemonitoring follow-up in reducing hospital readmissions, ED visits, and total hospital days for high-risk postdischarge patients. Our findings demonstrate that home digital monitoring significantly reduced hospitalizations, ED visits, and total hospital stay days at both 3 and 6 months after intervention.

The biggest obstacle in implementing discharge management for high-risk patients is the digital divide between patients and caregivers, and the degree of this divide varies significantly between urban areas and resource-limited regions. Most older adult individuals are unable to operate related digital home devices, and caregivers also have a lower acceptance of digital tools. Even if other family members living together have the ability to use digital devices, they often have to go out to work and cannot operate or transmit data during the day. Among the successfully enrolled cases, 46% (19/41) were aged 65 years or older. These older adult cases require remote home monitoring, allowing physicians to monitor their condition from home. The biggest factor comes from the assistance of younger family members who can complete most of the monitoring tasks. Among them, 12% (5/41) of the cases are aged 80 years or older, and they have a high willingness to self-monitor with the guidance provided. They can operate monitoring devices on their own and even record heart and lung sounds through video appointments with physicians. Therefore, although age may present a learning barrier, if there is support and encouragement from younger family members or a proactive attitude toward promoting health, remote health care can be a beneficial medical service for rural residents who lack sufficient medical resources [11,12].

Our study provides preliminary evidence suggesting that home digital monitoring may reduce hospital readmissions and ED visits among high-risk postdischarge patients. These findings align with previous research demonstrating the benefits of telehealth interventions in managing chronic conditions and improving patient outcomes. The study by Crowley et al [13] examined the effects of a comprehensive telehealth intervention compared with telemonitoring and care coordination in patients with persistently poor type 2 diabetes control. In this randomized clinical trial of 200 adults, the hemoglobin A_1c_ level improved by 1.59% at 12 months among those randomized to receive the comprehensive telehealth intervention, compared with a 0.98% improvement for the telemonitoring and care coordination group. While our study focused on a different patient population, the positive impact of remote monitoring and support on health care utilization and patient outcomes is consistent with their findings. These studies support the notion that digital health tools can play a crucial role in managing high-risk postdischarge patients, although the specific interventions and patient populations may vary.

The digital divide may also exist on the health care provider side. The operation of digital platforms and equipment requires learning and familiarity. People’s awareness of telemedicine centers and whether they are aware of the existence of such services on the health care side has been raised during outpatient education sessions. Some individuals have inquired about the long-standing implementation of telemedicine and why they were unaware of this health care resource. If telemedicine is implemented during outpatient hours, those in need can consult the telemedicine center, which would increase awareness of telemedicine and familiarity with the fixed members providing telemedicine, resulting in higher acceptance. However, if telemedicine is implemented in the ED, there may be pressure to complete tasks within limited timeframes due to the lack of fixed operators, and any difficulties in user experience could leave a negative impression [14,15]. In addition, in remote areas, grassroots health care providers are often required to use various digital tools at different times due to different programs, which is also a source of the digital divide.

The crucial value of digital health lies in the connection, including the connection between people and technology. In recent years, digital health care has flourished in Taiwan, with the government investing in and implementing telemedicine in mountainous and remote island areas, as well as promoting regional collaborative health care models in resource-limited regions. However, due to different regulatory and funding agencies, it is common to see various telemedicine services and platforms that cannot communicate with each other within the same county or city. The introduction of information and communication technologies is meant to connect needs and resources, and if incompatible platforms lead to barriers and resource waste, the original intention of connection is lost. As warned by WHO, the lack of integration and interoperability in the fragmented and disjointed digital health services, devices, and platforms pose greater challenges to resource-limited areas in need of assistance [16].

Therefore, when planning and investing in the infrastructure of digital health, the government and relevant agencies should establish a diverse and interoperable mechanism for telemedicine platforms in accordance with international information standards and security frameworks, within the framework of regulations and institutions. Digital tools and platforms should serve as mediums and channels for delivering health care content, rather than limitations. Building an integrated and interoperable ecosystem is essential to enhance the quality and accessibility of health care through digital means [17-19].

Several limitations should be considered when interpreting our findings. The study’s small sample size (41 participants) limits the generalizability of the results. In addition, the retrospective design and reliance on historical controls may introduce bias. The study also did not account for potential variations in individual patient adherence to the telemonitoring protocol, which could affect the outcomes. Furthermore, the digital divide, including differences in the ability of the patients to use technology and access to reliable internet, could impact the effectiveness of telemonitoring.

### Conclusion

Our study provides preliminary evidence that home telemonitoring can significantly reduce hospital readmissions and ED visits among high-risk postdischarge patients. These findings suggest that telemonitoring is a viable and scalable solution for improving postdischarge care and reducing health care utilization.

The broader implications of our findings are substantial. Telemonitoring can enhance patient outcomes, especially in underserved areas, by providing continuous health monitoring and timely medical interventions. This approach can also alleviate the burden on health care systems by reducing unnecessary hospitalizations and emergency visits, ultimately leading to cost savings. Future research should focus on larger, prospective studies to confirm these results and explore the potential of telemonitoring in diverse patient populations. Addressing the digital divide and enhancing system interoperability are crucial steps toward maximizing the benefits of telehealth interventions.

In conclusion, integrating advanced telemonitoring systems in postdischarge care for high-risk patients holds promise for improving health care delivery and patient outcomes. Our findings underscore the importance of adopting digital health tools to achieve better health management and resource optimization in health care systems.

## Appendix

Multimedia Appendix 1 Questionnaire of remote smart discharge management for high-risk discharged patients.

## Abbreviations

CAT: Chronic Obstructive Pulmonary Disease Assessment Test
COPD: chronic obstructive pulmonary disease
ED: emergency department
ICU: intensive care unit
IRB: Institutional Review Board
mMRC: modified Medical Research Council
WHO: World Health Organization

## Data Availability

All data generated or analyzed during this study are included in this published article and Multimedia Appendix 1.
