# Supplementary material for: Efficacy of Remote Health Monitoring in Reducing Hospital Readmissions Among High-Risk Postdischarge Patients: Prospective Cohort Study
Source: JMIR Form Res. 2024 Sep 13;8:e53455. doi: 10.2196/53455 (PMC11437225; doi:10.2196/53455)
Supplement: Multimedia Appendix 1 [file formative_v8i1e53455_app1.docx]

**National Taiwan University Hospital Yunlin Branch**

**Telemedicine Center and "2022 Remote Smart Discharge Management for High-Risk Discharged Patients"**

| **Digital Divide Questionnaire** |
| --- |
| **1. Gender:** □ Male □ Female  **2. Age:** □ 21-40 years □ 41-60 years □ 60-70 years □ 70-80 years □ 80 years and above  **3. Education Level:**  □ Unknown □ Elementary School □ Middle School □ University □ Higher than University  **4. 3C Exposure Level:**  □ Rarely use 3C products □ Use 3C products daily, such as smartphones □ 3C expert  **5. Experience with Operating Health Instruments:**  □ Yes, have health instruments at home: ___________ □ No  **6. Health Knowledge Reading:**  □ Do not specifically read  □ Watch medical-related TV programs  □ Only pay attention to health knowledge related to own disease  □ Regularly read health knowledge or journals  □ Others: ____________________________  **7. Self-operation:**  □ Yes **(skip questions 8-13)** □ No **(continue with questions 8-13)**  ------------------------------------------------------------------------------------------------------------------------------  **8. Assistant:**  □ Family members: wife, son, daughter, nephew/niece, grandchild □ Caregiver or foreign worker  **9. The person assisted with operation is:**  □ Family member □ Caregiver □ Nursing staff  **10. Education Level:**  □ Unknown □ Elementary School □ high school □ University □ Higher than University  **11. Assistant's Age:**  □ 21-40 years □ 41-60 years □ 60-70 years □ 70-80 years  **12. Exposure to Electronic Products:**  □ Rarely use 3C products □ Use smartphones □ 3C expert  **13. Ability to Operate Health Instruments:**  □ Already know how to use □ Learned to use after instruction  □ Do not know how to use and unwilling to learn |
| - **Instrument Usage Status** |
| **When operating the following instruments:**  **(1) Blood Pressure Monitor**  □ Very smooth, no difficulties at all □ A bit difficult □ Completely do not know how to operate  **(2) Pulse Oximeter**  □ Very smooth, no difficulties at all □ A bit difficult □ Completely do not know how to operate  **(3) Forehead Thermometer**  □ Very smooth, no difficulties at all □ A bit difficult □ Completely do not know how to operate  **(4) Glucometer**  □ Very smooth, no difficulties at all □ A bit difficult □ Completely do not know how to operate  **(5) Electrocardiogram (ECG)**  □ Very smooth, no difficulties at all □ A bit difficult □ Completely do not know how to operate  **When having difficulties operating the instruments:**  □ (1) Ask nursing staff or research assistant  □ (2) Use built-in resources, such as instrument instructions  □ (3) Give up using  □ (4) Other assistance methods: __________________________________________________________  ________________________________________________________________________________ |
| - **Tablet Usage Status** |
| 1. Usage of tablet's built-in assistance information or software:   □ No  □ Yes, used   1. Clarity of assistance information or software interface:   □ Very clear  □ Not clear, text too small, or difficult to understand   1. Was the assistance information helpful to you?   □ Yes  □ No   1. Are you satisfied with the experience of using this care package?   □ Very satisfied  □ Satisfied  □ Okay  □ Not satisfied   1. Which instrument in the care package do you like?   ______________________________________________________________________________  Why: ______________________________________________________________________________   1. Which instrument in the care package do you dislike?   ______________________________________________________________________________  Why: ______________________________________________________________________________ |
| 4 .Other suggestions: |
| Survey completed. Thank you for your cooperation! |
| After conducting the survey, the conclusions are as follows: |
| 1.Measurement period: ________________, totaling ___ weeks, with records for _____ days.  2.Types of recorded data:  □ One type □ Two types □ Three types □ Four types □ Five types □ Six types  3.Achievement rate of measurements: __________% |
